# Characterization and Modeling of Nano Wear for Molybdenum-Based Lubrication Layer Systems

**DOI:** 10.3390/nano11061363

**Published:** 2021-05-21

**Authors:** Bernd-Arno Behrens, Gerhard Poll, Kai Möhwald, Simon Schöler, Florian Pape, Dennis Konopka, Kai Brunotte, Hendrik Wester, Sebastian Richter, Norman Heimes

**Affiliations:** 1 Institute of Forming Technology and Machines, Leibniz Universität Hannover, An der Universität 2, 30823 Garbsen, Germany; behrens@ifum.uni-hannover.de (B.-A.B.); brunotte@ifum.uni-hannover.de (K.B.); wester@ifum.uni-hannover.de (H.W.); srichter@ifum.uni-hannover.de (S.R.); 2 Institute for Machine Design and Tribology, Leibniz Universität Hannover, An der Universität 1, 30823 Garbsen, Germany; poll@imkt.uni-hannover.de (G.P.); pape@imkt.uni-hannover.de (F.P.); konopka@imkt.uni-hannover.de (D.K.); 3 Institute for Materials Science, Leibniz Universität Hannover, An der Universität 2, 30823 Garbsen, Germany; moehwald@iw.uni-hannover.de (K.M.); schoeler@iw.uni-hannover.de (S.S.)

**Keywords:** nano indentation, CoF, nano wear, SPM, wear behavior, micro tribology, Mo-coatings, wear modelling

## Abstract

As a result of global economic and environmental change, the demand for innovative, environmentally-friendly technologies is increasing. Employing solid lubricants in rolling contacts can reduce the use of environmentally harmful greases and oils. The aim of the current research was the development of a solid lubricant system with regenerative properties. The layer system consisted of a molybdenum (Mo) reservoir and a top layer of molybdenum trioxide (MoO_3_). After surface wear, Mo is supposed to react with atmospheric oxygen and form a new oxide. The determination of the wear volume of thin layers cannot be measured microscopically, which is why the wear behavior is initially determined on the nano level. In this work, single Mo and MoO_3_ coatings prepared by physical vapor deposition (PVD) are characterized by nano testing. The main objective was to determine the wear volume of the single coatings using a newly developed method considering the initial topology. For this purpose, nano-wear tests with different wear paths and normal forces were carried out and measured by in situ scanning probe microscopy (SPM). Based on the characteristic values determined, the coefficient of wear was determined for wear modeling according to Sarkar. The validation of the wear model developed was carried out by further wear tests on the respective mono layers.

## 1. Introduction

**Figure 1:**
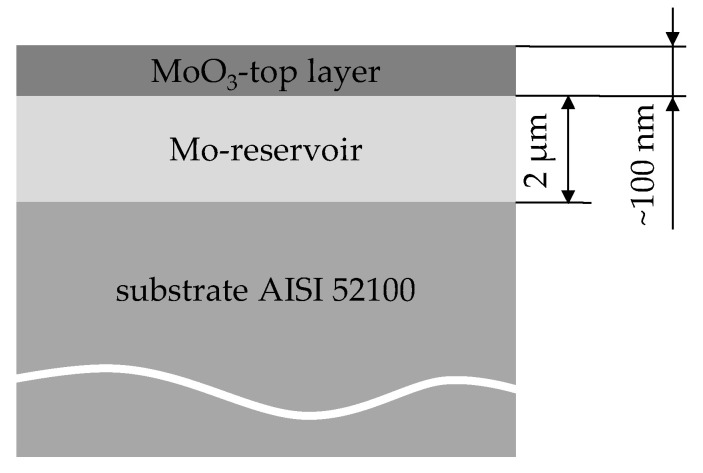
Schematic structure of the self-regenerative molybdenum layer system.

Solid lubricants show high potential substitutes for the use of greases and oils, so that the application of environmentally harmful lubricants can be minimized [1]. Another advantage of solid lubricants is that they are used directly in tribological contact areas so that the entire component or system does not have to be wetted with oil or grease. This means that the resources used are applied in a targeted manner. Molybdenum coatings have been proven to be particularly effective as solid lubricants. For example, molybdenum disulfide (MoS_2_) is used as a solid lubricant for rolling bearings, which minimizes the friction values [1]. However, the application of pure solid lubricants is limited because they are usually not wear-resistant and quickly exhausted. Therefore, a layer system was developed in which the solid lubricant is continuously reformed. The considered self-regenerative molybdenum layer system was built up as shown in Figure 1. As the substrate, the hardened bearing steel AISI 52100 was used, on which a 2 µm thick molybdenum (Mo) reservoir was sputtered using the physical vapor deposition (PVD) process [2]. The top layer of the system was molybdenum trioxide (MoO_3_), which was applied as a very thin layer of approximately 100 nm. The MoO_3_ ensures that the solid lubricant is already available in the running-in phase. As soon as the top layer is worn away, a new oxide layer is regenerated from the Mo-reservoir by tribo-oxidation. In addition to the contact pressure, the contact temperature, and the surrounding media, the contact surface is also decisive for tribo-oxidation [3,4,5,6].

To describe abrasive wear mathematically, the Archard approach is often applied to predict the wear volume. Archard established that the wear volume *w* can be calculated as a function of the normal force *F*_N_, the material hardness *H*, and the sliding distance *s*. Since the wear volume also depends on the contact bodies, a coefficient of wear *k* is also required [7]. The Archard model was extended by Sarkar to include the occurring tribological conditions in the contact. For this purpose, a friction term was introduced to describe the friction between the contact partners [8]. The Archard wear model extended by Sakar is described in Equation (1).

*w* = *k* · (*F*_N_ · *s* · (1 + 3*µ*^2^)^0.5^)/*H* (1)

For the parameterization of wear models, wear tests are carried out under known boundary conditions and the wear volume is determined using optical images or weight measurements [9,10,11]. These methods of volume determination are limited with respect to thin layers such as the MoO_3_ top layer. For this reason, there is already a wide variety of methods that characterize wear on the nano scale. On the nano and micro levels, wear tests are not standardized and differ in terms of test forces, test paths, and procedures. In terms of test forces, scanning probe microscopy (SPM) includes atomic force microscopy (AFM) or frictional force microscopy (FFM) and is mainly used for the nano level [12,13,14,15]. In this context, many investigations of nano wear using AFM and FFM have been carried out on polymers [16,17,18,19,20,21,22]. Investigations on metals are not very common, but some investigations have been carried out on gold, ferrite, and stainless steel [23,24,25,26,27]. Regardless of the material used, wear tests, scratching, and sliding tests have been carried out, characterizing line wear and, among other effects, coating failure. The wear volume was determined by means of individual profile scans or by combining several profile scans [28,29,30,31,32]. In the conventional wear test, a wear field is generated with a constant load and through multiple passes. The wear volume is determined using different approaches. The calculation of wear volume using 2D profiles through the wear surface was used in [17,18,24]. In addition to wear tests with constant force, tests with increasing force within a wear field were also carried out [19]. The studies presented so far all determine the wear volume without taking into account the condition of the surface before the wear test. The work of Wang et al. showed the great influence neglecting the initial condition can have on the wear volume determined [33]. They considered the surface structure before the wear test by means of pre-scan and post-scan. Since the crystal in the scan head causes offsets between the measurements due to the non-linear piezoelectric effect, the offsets must be compensated by selecting unworn surfaces for the determination of the offset [33]. However, this method of determining the offset highly depends on the surface quality and the quality of the scan. In addition, a unified evaluation is difficult.

The contact temperature on the nano level is the same as the ambient temperature as the speeds and contact areas are small. Due to the small contact areas, very high contact pressures of several GPa occur on the nano level, even with the smallest loads [34]. It must be investigated whether the boundary conditions on the nano level are sufficient to form an oxide layer in the wear surface or whether wear particles are enriched with oxygen and act as a transfer lubricant. The Mo-reservoir has to be more wear-resistant than the MoO_3_ top layer, for example, a higher hardness or strength in order to minimize wear in the contact surface [35,36,37].

In this work, the extended wear model according to Sarkar is parameterized on the nano level. For this purpose, the hardness of the layers and the coefficients of friction (CoF) were determined. To determine the wear volumes, a new unified method for calculating the wear volume using in situ SPM imaging is presented. For this purpose, targeted reference indents are applied, with which it is possible to find out the offset between the scans and to automate the evaluation of the pre-scan and post-scan. By varying the normal forces and the wear paths, the coefficients of wear (CoW) for the Mo and MoO_3_ layers can be determined. The CoW determined for the coatings were the basis for the wear modeling by Sarkar. Finally, the models developed were experimentally validated by further wear tests. In addition to the characterization and parameterization of the wear models, the wear fields were analyzed using scanning electron microscopy (SEM) in order to obtain information on the wear behavior and tribo-oxidation.

## 2. Materials and Methods

### 2.1. Manufacture of the Specimens

In terms of a reproducible and successful deposition of the coating on the steel substrate surface, the experience gained from previous investigations was taken into account [2,34,35]. Hardened rolling bearing steel AISI 52100 was used as the substrate for the coatings as cylindrical plates with a diameter of 14 mm and a height of 5 mm. The surface of the steel substrate plates was ground plane-parallel and then polished to a mirror finish. Before sputtering, the specimens were cleaned in an ultrasonic bath for 15 min with acetone and isopropanol. A vacuum-based PVD process was used to deposit molybdenum and molybdenum trioxide. After setting the vacuum of 6 × 10^−7^ mbar in the recipient, the specimens were first cleaned with a material-removing plasma etching process. The etching was performed for a time of 5 min. The steel substrate was heated to a steady-state temperature of 200 °C and the recipient was filled with argon at a flow rate of 50 sccm. A Mo target was used for the molybdenum coating and a MoO_3_ target was used for the molybdenum trioxide layer to deposit the individual coatings on the steel substrate surface, respectively. A power density of 1.17 W/cm² was set for the deposition and the partial process pressure of argon during the coating was set to 2.2 × 10^−2^ mbar using a mass flow controller. The sputter time for the molybdenum was 60 min and for the molybdenum trioxide, it was 12 min. Using silicon wafers, the layer thickness could be determined in the scanning electron microscope. A layer thickness of 2 µm was determined for molybdenum and a layer thickness of 500 nm for molybdenum trioxide.

### 2.2. Nanotechnical Investigations

The investigations were carried out on a Hysitron TriboIndenter^®^ TI 950 (Bruker, Minneapolis, MN, USA) with a range of possible forces from 2 μN to 10,000 μN. For this purpose, a capacitive three-plate transducer (2D transducer) was used in combination with a high-precision 3-axis tandem tube piezo scanner. All tests were carried out under laboratory conditions, with an ambient temperature of 17 °C ± 1 °C and a humidity of 38% ± 6%. A triangular Berkovich diamond tip with a tip radius of less than 50 nm was used to determine the nano hardness and the modulus of elasticity. The indentation was force-controlled via a trapezoidal force-displacement function to avoid dynamic measurement defects [38,39]. The calibration of the Berkovich tip was carried out on a fused quartz specimen in the range from 100 µN to 10,000 µN. An area function was determined, which requires a minimum penetration depth of 50 nm. In the nanotechnological testing of coatings, it is particularly important to make sure that measurements on the surface are not influenced by the substrate. The 10% rule has proven to be useful when the coating is harder than the substrate [38]. Following the 10% rule, only the coating is characterized if the penetration depth is max. 10% of the coating thickness. If the coating is softer than the substrate, the 30% rule can be applied [38,40]. To determine the coating hardness, the test forces were varied to determine at which force the minimum penetration depth of 50 nm was exceeded. The steel substrate hardness is known from previous investigations [34], so that the layer hardness can be determined by exceeding the minimum penetration depth and it can be decided which of the two rules must be used. By analyzing the resulting penetration depth, the process parameters for the hardness measurement were determined. For each test load, 50 indents were performed with a spacing of 8 µm in order to obtain a statistical validation.

**Figure 2:**
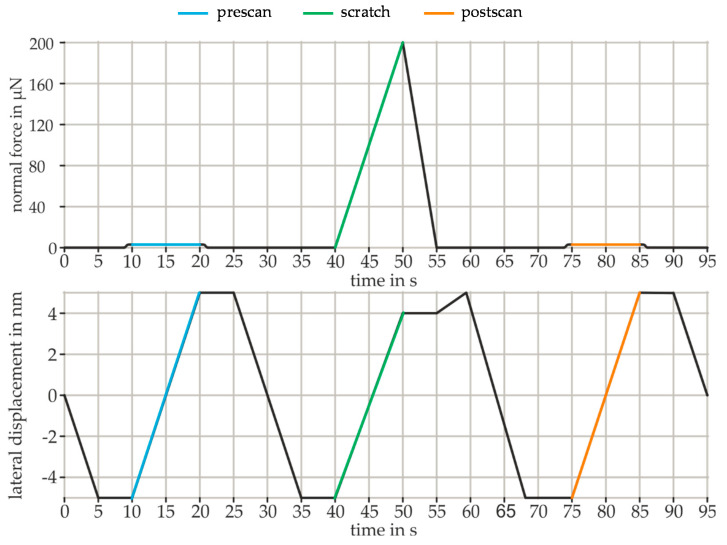
Chronological sequence of normal force and lateral displacement for the scratch test.

To investigate the elastic and plastic material behavior, scratch tests were used. The CoF were determined by recording the resulting lateral force over the scratch length. For the scratch tests, a conical diamond tip with a tip radius of 300 nm was used. A conical tip has no preferred scratching direction, which facilitates installation and calibration and makes the results more comparable. The chronological sequence of the scratch test for the specified course of the normal force and the lateral displacement is shown in Figure 2. First, the lateral distance from −5 µm to 5 µm is recorded with a constant scanning force of 2 µN in the pre-scan (blue line). During the following scratching process, the normal force was continuously increased to the specified maximum (green line). After reaching the maximum load at 4 µm, the tip was unloaded without any further lateral displacement. This ensures that the spring is not overstressed and the tip moves forward. The tip then moves in the unloaded state (55 s–60 s) until the end of the test section at 5 µm. In the post-scan, the normal force is again kept constant at 2 µN in order to determine the remaining plastic deformation (orange line). The normal forces were based on the standard forces provided for the wear tests 50 µN, 100 µN, 150 µN, and 200 µN. In addition, scratch tests were carried out at 1000 µN normal force in order to investigate how the elastic and plastic material behavior changes at higher loads and how the CoF is affected. Each scratch test was repeated eight times for statistical verification.

**Figure 3:**
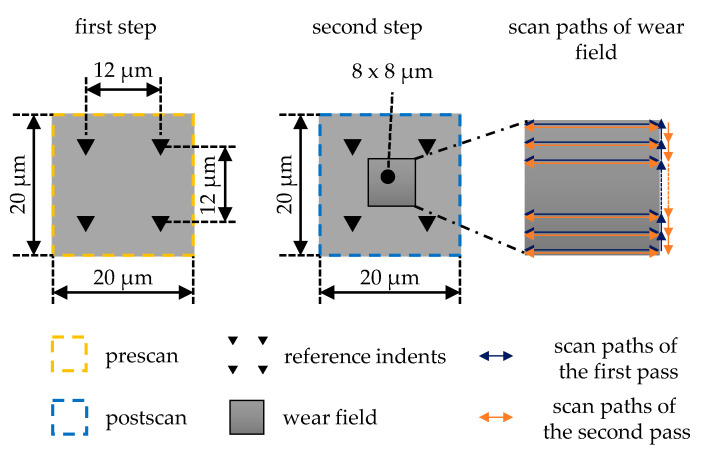
Schematic representation of the procedure of the extended wear test.

The conventional wear test was carried out using in situ SPM. The wear field was scanned according to the specified parameters such as scan size, scan rate, number of passes, normal force, and the number of lines for scanning, and then the measuring location is directly scanned over a large area using a post-scan. The nano wear test method developed and used here is an extension of the conventional wear test and is illustrated graphically in Figure 3. For this purpose, the wear test was divided into two steps. In the first step, four reference indents were placed at a distance of 12 µm around the measuring location. Then, the measuring location was scanned with the reference indents to determine the initial condition of the measuring location before the wear test in the course of the so-called pre-scan. The second step begins with the wear test, which was placed centrally between the reference indents. The size of the wear field was 8 µm × 8 µm. The wear field was built up line by line. Here, it is necessary to consider that the same line was used for the forward and backward path and that the tip is subsequently moved vertically to pick up the next line. The fact that the lines have been moved twice is particularly important for later wear modeling when the total wear distance has to be determined. When all 512 lines have been recorded, the first pass (blue) is completed and the second pass begins (orange). The tip remains in contact with the specimen during the entire wear test. After termination of the specified number of passes, the post-scan was taken from the measuring location including wear field and reference indents.

For the nano wear test, a cube corner indenter made of diamond with a tip radius less than 50 nm was used. The cube corner is a very sharp-edged indenter, which can cause wear even on very hard materials. Another reason for using the cube corner indenter is that it affects less volume during indentation than, for example, the Berkovich or a conical indenter. Thus, the reference indents can be positioned close to the wear field without influencing the wear behavior of the coating. Furthermore, the penetration depth of the cube corner indenter is significantly greater than that of the Berkovich or conical indenter, even at moderate loads, so that it is much easier to detect the reference indents as the deepest points later on. A force of 8000 µN was used for the reference indents in the Mo-coating and a force of 4000 µN for the MoO_3_-coating. The lower force for the MoO_3_ is due to the fact that the greater loads resulted in a significantly greater pile-up, which in some cases came very close to the wear field. Since a sufficient penetration depth was achieved with 4000 µN and at the same time the pile-up could be reduced, the force was used for the reference indents. Table 1 lists the settings for the pre-scan, the post-scan, and the wear field. The scan rate determines the speed of the scanned image, and slower scan rates will offer less image distortion while higher scan rates may cause noise or artifacts depending on the specimen. To ensure that the resolution and quality of pre-scan and post-scan were as high as possible, the scan rate was reduced to 0.5 Hz, and a minimum scanning force of 2 µN was set so that no material was removed during the scans. Beake et al. investigated the influence of the number of lines on the scan quality and showed that the scan quality could be significantly increased with an increasing number of lines [16]. Therefore, the number of lines was set to 512, as this is a good compromise between scan quality and scan time. For the wear field, the scan rate was set as constant to 1 Hz. Koinkar et al. also found that the scan rate did not have a large influence on the wear behavior on the nano level [41], whereas Beake et al. and Degiampietro et al. found an influence of the scan rate on the wear behavior [16,24].

The test matrix considering the variations and combinations of scanning force and number of passes are listed in Table 2. For each combination of force and number of passes, four repetitions were performed, each at two different locations on the specimens. This is of great importance since the wear tests reflect the wear behavior of the coating very locally. For the modeling of the wear coefficient, the parameter combinations highlighted in grey were used. In order to validate the model, further parameter combinations were tested that lie within the limits of the model, highlighted in orange.

### 2.3. Wear Modeling

The basic requirement for parameterizing the Sarkar wear model is the exact determination of the wear volume. The procedure for determining the wear volume applied in the scope of this work is described in detail below.

**Figure 4:**
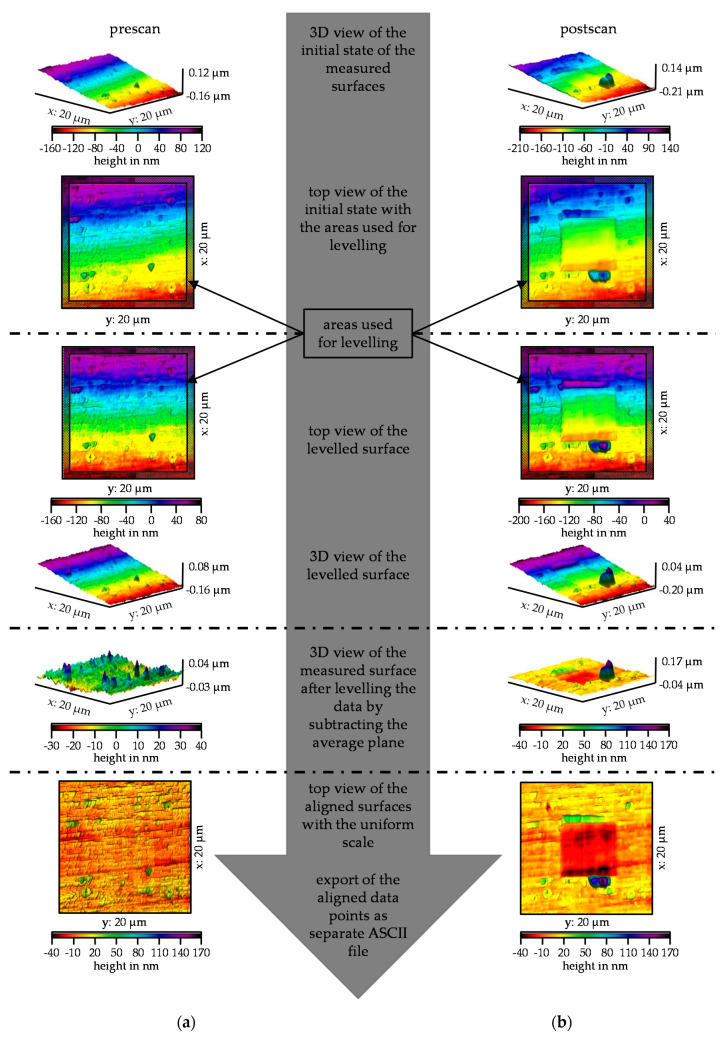
Gwyddion^®^ workflow of the standardized transformation steps and their effects on the surface of (**a**) The pre-scan and (**b**) The post-scan on the MoO_3_ specimen.

The result of a wear test is a pre-scan and a post-scan. The scans were generated via in situ SPM imaging, whereby one image is generated on the forward and one on the backward path. In addition, a superimposed image from the forward and backward paths was stored. The superimposed image reflects the surface in a distorted way, as the forward and backward paths show slight differences. For the evaluation of the volume, the scattered data taken on the forward path were used. This enables a comparison between the individual wear tests. Surfaces that are measured tactilely using in situ SPM imaging are always subject to tilting, since on the one hand, the specimens in this size range can never be plane-parallel and on the other hand, a curvature is always induced by the piezo scanner. This curvature was corrected by transformations so that the surfaces were available without tilting. To ensure that the recorded surfaces could be transformed uniformly, they were transformed in Gwyddion^®^ (version 2.56, open source software) using a defined routine. The individual transformation steps and their effects on the surface are shown in Figure 4 using the example of a pre-scan in (a) and a post-scan in (b) of MoO_3_. In the initial state, both scans were tilted about the x- and the y-axis. The tilt around the y-axis was greater than the tilt around the x-axis. This tilt was smaller, but still recognizable by the gap to the y-axis and the diagonal color gradient in the top view. For the first transformation step, the tilt around the x-axis was compensated. For this purpose, the edge areas of the scans were used, each comprising 34 lines. The levelling was done by an intersection of lines of the overlapping areas. This compensates for the tilt around the x-axis, which is now noticeable in the color gradient running parallel to the y-axis. Since the data were now only tilted around one axis, the levelling was compensated by subtracting the average plane. The data of the pre- and post-scan were now within the x–y plane, but still with different color scaling. By adjusting the color scaling, it became clear that the unworn areas of both scans were on a similar level. The unworn areas were not completely identical but showed a slight offset in the z-direction, which must be corrected in the subsequent MATLAB^®^ (R2020a, The MathWorks, Natick, MA, USA) evaluation. Therefore, the transformed surfaces were imported in MATLAB^®^ as ASCII files.

**Figure 5:**
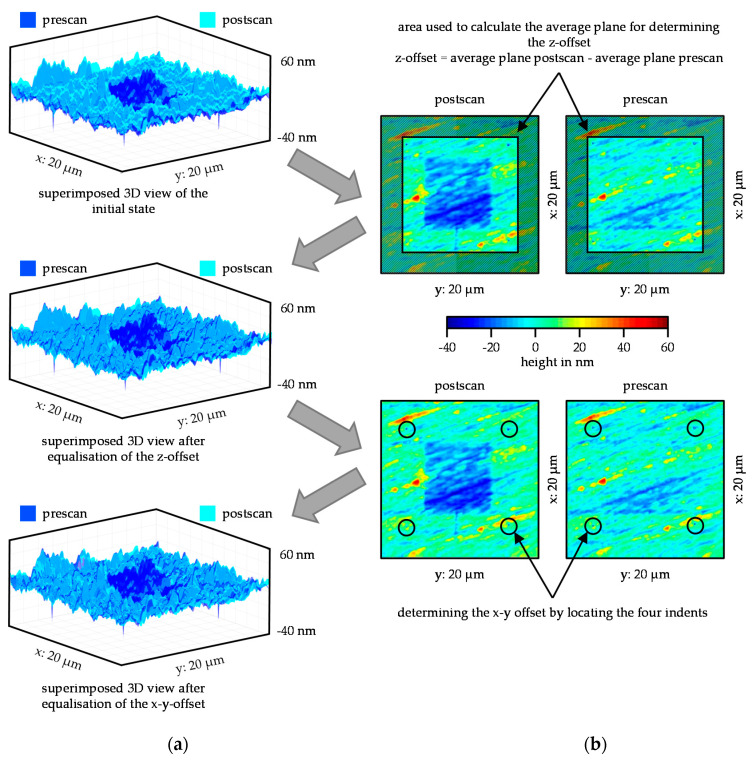
(**a**) Superimposed 3D plots of the pre-scan and post-scan, initial state (top), after correction (middle) of the z-offset, after correction of the x-y-offset (bottom). (**b**) Top view of pre-scan and post-scan to illustrate which areas were used for offset determination.

In Figure 5, the determination of the offset (a) and its effect on the pre-scan and post-scan (b) in MATLAB^®^ are shown. The top overlaying 3D plot shows the position of the data extracted from the ASCII files from Gwyddion^®^. The post-scan indicates slightly larger values in the z-direction than that of the pre-scan, which can also be seen in the export step in Figure 4. The z-offset is made up of two parts. First, the offset in z-direction was caused by the fact that the in-situ SPM imaging data was not subject to a global, but a local coordinate system. This means that if the same measuring point is scanned twice and the tip has to touch the surface again between the two scans, the values in z-direction differ from each other. The second part was based on the fact that the average plane was different in the post-scan than in the pre-scan due to the wear field. The subtraction of the data with the average plane results in smaller deviations in z-direction. This offset was corrected in MATLAB^®^ as follows. First, an average plane was calculated for the pre-scan and the post-scan from the unworn edge areas. The z-offset was determined by subtracting the average planes. Subsequently, each value of the pre-scan was shifted by the calculated z-offset. The middle 3D plot shows that the distance between pre-scan and post-scan had been significantly minimized. Nevertheless, the pre-scan did not completely match the post-scan. A closer look revealed that there was also an offset in the x- and y-direction. This offset also resulted from the re-detection of the surface between the two steps of the wear test. However, it is possible to determine the offset in x- and y-direction using the four reference indents. To do this, the four lowest points within the matrix were searched and the corresponding x- and y-coordinates were stored in a vector. The offset could be calculated from the two vectors of the coordinates. The pre-scan and post-scan were then shifted in x- and y-direction using the calculated offset. The result of the shift in x- and y-direction can be seen in the lower 3D plot in Figure 5, which showed a very homogeneous intersection of pre-scan and post-scan in the unworn areas.

**Figure 6:**
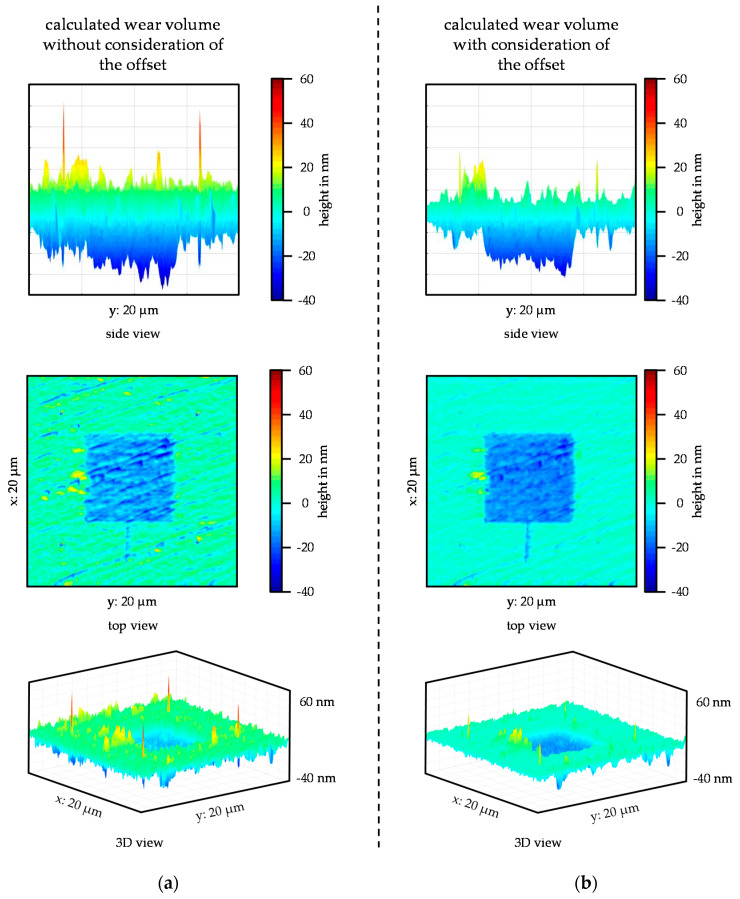
Results of the subtraction of the post- and pre-scan: (**a**) Without consideration of the offset; (**b**) With consideration of the offset.

By transforming and correcting the offsets, the pre-scan can now be subtracted from the post-scan. To illustrate the influence of the offset on the subtraction of post- and pre-scan, the results of the subtraction are shown in Figure 6a without considering the offset, and in Figure 6b considering the offset. In the side view in Figure 6a, a larger scatter than in (b) can be clearly seen. In addition, without considering the offset, it is difficult to separate the unworn areas from the wear field. Since the reference indents represent the lowest points, they have a particularly strong effect during subtraction in both directions if the indents are not exactly on top of each other. This shows the influence of the x- and y-offset, especially through the positive and negative peaks. The wear field during subtraction, under consideration of the offset, can be clearly seen in the side view. The height of the unworn areas was almost zero. However, smaller positive deflections of the reference indents could also be seen, which means that it can be assumed that an offset was still present here. The positive deflections of the reference indents and the other smaller differences in the unworn areas were not due to a further offset, but to the influence of the already known offset on the pre-scan and post-scan. If there was no offset between the pre-scan and post-scan, they would be identical in the unworn areas and both would show the same depth and position for the reference indents. However, if there is an offset between the two scans, there will automatically be deviations. If, for example, a line from the pre-scan records the deepest point of a reference indent, then an offset of a few nanometers in the post-scan means that it cannot record the same depth for this reference indent because the line of the post-scan features an offset. This situation causes a certain basic error, which cannot be eliminated, even by considering the offset. However, this error is significantly smaller than the error that occurs when the offset is not considered, or only the post-scan is evaluated without considering the initial surface of the pre-scan. It is possible that deviations may occur in the areas around the wear field. These deviations can be caused, for example, by material throw-ups or by a possible delamination of the layer. In addition, it is also possible that wear particles are stuck at the edges of the wear field.

**Figure 7:**
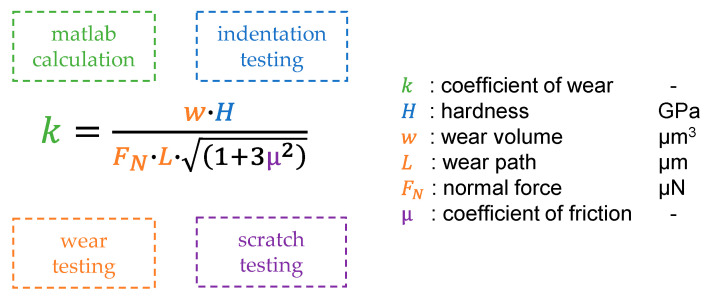
Calculation of the coefficient of wear with the assignment of the parameters to the test methods.

To calculate the wear volume, the wear field determined from the subtraction of the post and pre-scan was integrated. The integration was done using a trapezoidal function. However, it was not integrated over the entire wear field size of 8 µm × 8 µm, but over a field of 7 µm × 7 µm, which lies at the center of the wear field. The reason for reducing the size of the field for the integration was to avoid an influence of the edge areas on the integration. On one hand, material piles up in the edge area and on the other hand, errors in the detection of the correct transition were to be expected between the wear field and the unworn surface due to the geometry of the indenter. By determining the wear volume, the wear coefficients can be modeled. Figure 7 lists the various parameters needed to calculate the CoW and the measurement data on which they were based. The hardness of the respective layer was determined via nano indentation and the mean value of the layer hardness was used in the model. The CoF was determined from the scratch tests and considered over the various loads. The wear volume was averaged from the respective repeat tests and the average wear volume was used to calculate the CoW. Wear volume, normal force, and wear path were extracted from the wear tests. It must be taken into account that due to the field reduction in the integrations of the wear volume, the wear path must also be reduced accordingly, otherwise the CoW will be incorrect. As a result of the modeling, CoW are available as a function of normal force and wear path for the respective coating. By interpolation between the grid points of the test matrix, it is possible to calculate the wear coefficient for other parameter combinations and thus predict the wear volume.

### 2.4. SEM and Optical Investigations

The specimens were analyzed by SEM with energy-dispersive x-ray measurements (EDS) using a Supra 40VP (Zeiss, Oberkochen, Germany) to investigate the coatings and the characteristics of the wear fields. The main aim was to use Inlens images to prove that particles had become detached from the wear field during the nano wear test. Furthermore, line scans and element mappings were used to investigate the wear fields in order to prove the mechanism of tribo-oxidation and the formation of a possible oxide, especially from the molybdenum reservoir. In addition, the measuring points before and after the wear test were recorded with the light microscopic optics of the Hysitron TriboIndenter^®^ TI 950 (Bruker, Minneapolis, MN, USA) in order to gain initial information about the wear behavior.

## 3. Results and Discussions

### 3.1. Nano Indentation and Scratch Test

**Figure 8:**
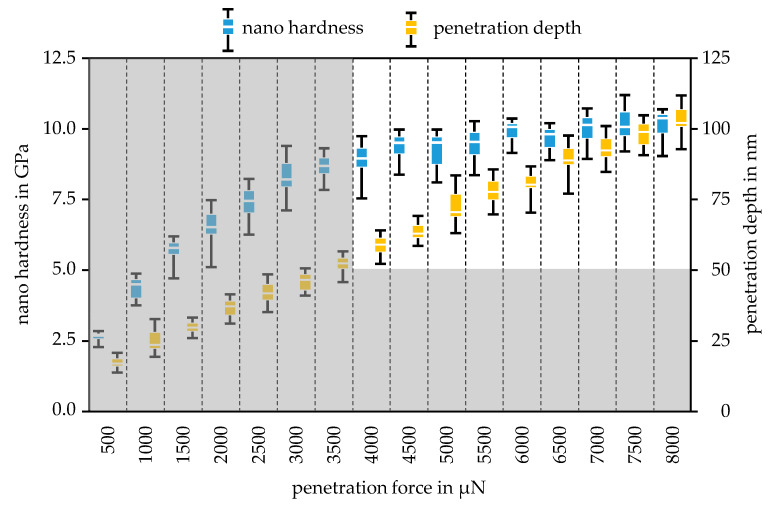
Development of the nano hardness and the penetration depth via the varied penetration forces for the Mo-coating with minimum and maximum values.

**Figure 9:**
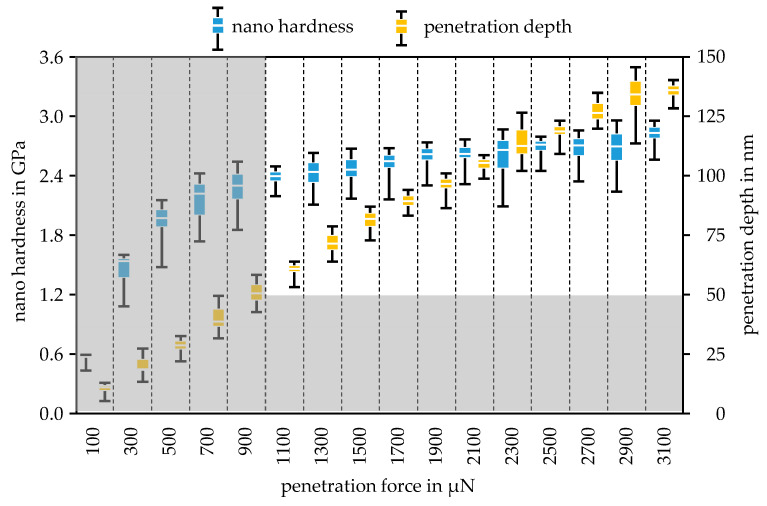
Development of the nano hardness and the penetration depth via the varied penetration forces for the MoO_3_-coating with minimum and maximum values.

The development of the nano hardness in dependence of the increase in penetration force was plotted for the Mo-coating in Figure 8 and for the MoO_3_-coating in Figure 9. The error bars of the boxplots represent the minimum and maximum values of the respective measurement series. Since the Berkovich tip was calibrated for a minimum penetration depth of 50 nm, all measurements with a penetration depth below 50 nm were erroneous. Measurements below the minimum penetration depth are greyed out. For the Mo specimen, the hardness can be determined with forces between 4000 µN and 8000 µN.

The average value of the Mo hardness in this force range is 9.82 GPa, which is higher than the steel substrate hardness of 9.30 GPa. Considering the standard deviation of the hardness measurements for Mo and the steel substrate listed in Table 3, it can be seen that the Mo hardness is approximately equal to the hardness of the steel substrate. Thus, the maximum penetration depth can be determined with the 10% rule. The maximum penetration depth of 110 nm at 8000 µN clearly shows that the 10% rule was fulfilled for a Mo layer thickness of 2 µm. In this range, the hardness values were almost constant, although the penetration depth increased continuously.

This circumstance and the compliance with the minimum penetration depth as well as the fulfilment of the 10% rule allow for the conclusion that the measured hardness reflects the pure hardness of the coating. For the MoO_3_-coating, the layer hardness was measured in the force range from 1100 µN to 3100 µN. In this case, the hardness values also remained along a constant level over the different forces. Since the MoO_3_ layer is significantly softer than the steel substrate, the 30% can be applied. With a coating thickness of 500 nm, a penetration depth of 150 nm would be permissible, which was not exceeded in the force range considered, but was almost reached with 2900 µN and 3100 µN, so that these forces represent the upper limit for characterization. The averaged hardness of the coating is listed in Table 3, which was used for the subsequent parameterizing of the wear model. In Table 3, the modulus of elasticity of the Mo and the MoO_3_ are listed. The Young’s modulus of Mo was more than three times that of MoO_3_. Due to the equally large differences in nano hardness, this is a plausible result, which was analyzed by examining the elastic and plastic material behavior in the scratch test.

**Figure 10:**
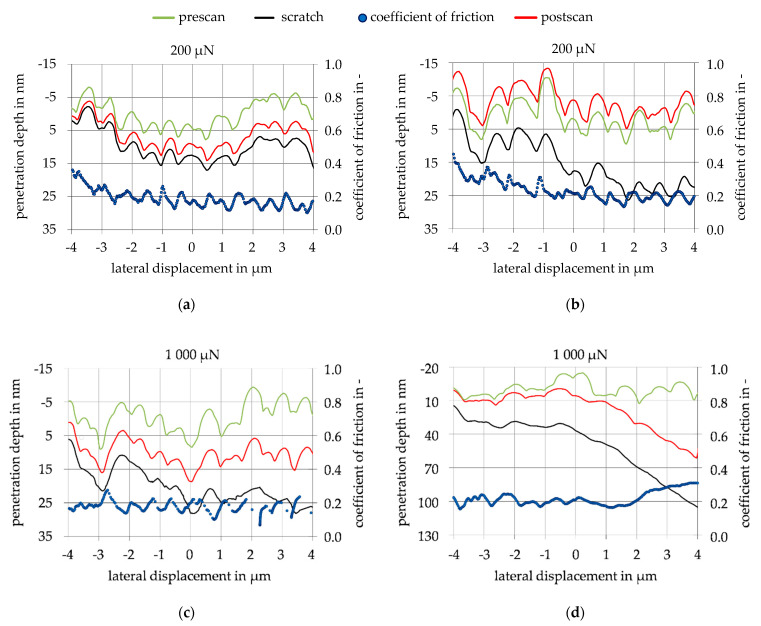
Resulting penetration depth of pre-scan, scratch, post-scan, and coefficient of friction: (**a**) Normal force of 200 µN for the Mo-coating; (**b**) Normal force of 200 µN for the MoO_3_-coating; (**c**) Normal force of 1000 µN for the Mo-coating; (**d**) Normal force of 1000 µN for the MoO_3_-coating.

Figure 10 shows an example of the results of the scratch test for both coatings. The CoF was recorded over the complete scratch path. After a running-in period, the CoF stabilizes and shows slight fluctuations due to the surface condition. A CoF determination at the end of the scratch at 4 µm therefore always depends on the surface, hence the CoF was determined from the lateral displacement of 3 µm and 4 µm and an average value was formed. Over the investigated normal forces of 50 µN to 200 µN, there were no significant changes in the CoF for the respective coating, which can also be seen in the low standard deviation of the CoF in Table 3. For the CoF of the Mo-coating, an average friction value of *µ* = 0.13 and for MoO_3_, an average friction value of *µ* = 0.20 was achieved. These CoF were used to parameterize the wear model. The elastic material behavior is described by the difference between the penetration depth of the scratch and the post-scan. The plastic deformation is determined by the difference between pre-scan and post-scan. The results of the scratch test for a normal force of 200 µN are shown in Figure 10a for the Mo-coating and in Figure 10b for the MoO_3_-coating. When comparing the two scratches, it was noticeable that the elastic behavior of the MoO_3_-coating was significantly greater than that of the Mo-coating. This reaction did not correlate with the measured Young’s modulus. Due to the low Young’s modulus of MoO_3_, a larger proportion of plastic behavior and a smaller proportion of elastic behavior would have been expected in the scratch test. Comparing the scratch test with a normal force of 1000 µN in Figure 10c,d, it can be seen that the material performance of Mo was similar to the performance at 200 µN normal force. The material behavior of MoO_3_, however, changed over the lateral displacement due to the increasing normal force. With a lateral displacement in the range from −4 µm to 1 µm, the response was similar to the scratch in Figure 10b, with a very large elastic portion. The behavior changed over the lateral displacement from 1 µm to the end of the scratch at 4 µm. There, the plastic portion increased significantly and corresponded to the behavior expected in this section. The CoF in Figure 10d also increased to *µ* = 0.3 due to the changed material behavior, whereas the CoF in Figure 10c reached the same CoF as in Figure 10a. This change in the material behavior of MoO_3_ can be explained by near surface compressive stresses, which were only exceeded at a greater load and penetration depth. The normal force at which the change in material behavior occurs is 650 µN. However, this force is clearly outside the test matrix, so it is important to investigate how the MoO_3_ layer behaves in the wear test of repeated loading with a lower normal force.

### 3.2. Wear Tests

**Figure 11:**
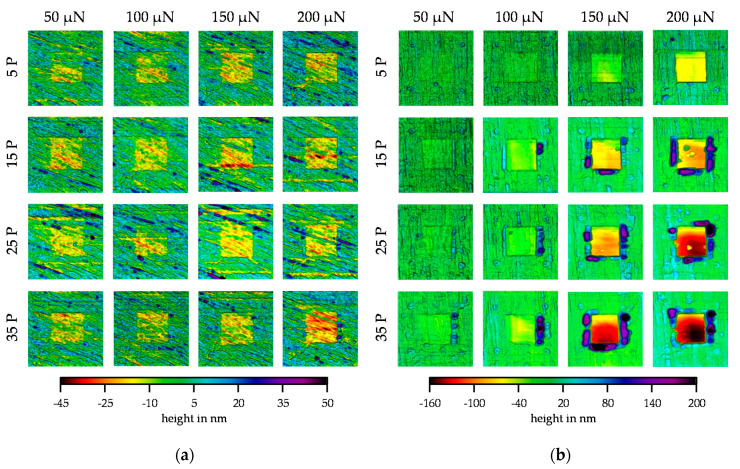
Evolution of the wear fields over the normal forces (µN) and number of passes (P) investigated: (**a**) Mo-coating; (**b**) MoO_3_-coating.

The post-scans taken by in situ SPM imaging for the combinations of number of passes (P) and investigated normal forces are shown in Figure 11a for the Mo-coating and in Figure 11b for the MoO_3_-coating. With increasing normal force and number of passes, the post-scans in Figure 11a showed a more pronounced wear field. The wear depth increased equally with increasing normal force and number of passes so that a linear wear behavior can be assumed. However, the wear surfaces still had the structure of the unworn surface so that there was no complete levelling of the structure. In addition, no material build-ups could be seen at the edges of the wear area. In the post-scans of MoO_3_, on the other hand, a clearly different wear behavior can be seen in Figure 11b. With increasing normal force and number of passes, the wear depth also increased. However, the wear depth did not increase linearly, but exponentially, starting at a normal force of 150 µN. It was also noticeable that the surface structure in the wear fields could only be recognized in the fields with a load of 50 µN and in the fields with 5 P up to a force of 100 µN. Particularly in the ranges of 50 µN/5 P, 50 µN/15 P, and 100 µN/5 P, the structure was still well visible. The lower wear depth of these parameter combinations may have been influenced by the possible near-surface compressive stresses as compressive stresses can have a wear-reducing effect. Furthermore, the low wear depth reflects the elastic behavior from the scratch test. The other fields showed a greater depth of wear, so that the possible near-surface compressive stresses were reduced by the repetitive loading, and thus greater wear occurred. In these wear areas, the surface structure was completely levelled. Furthermore, it was noticeable that above 15 P and above a force of 100 µN, there was a strong build-up of material at the edges of the wear fields, which increased, especially with an increase in force. Due to the levelling of the wear surfaces and the increased material build-up, abrasive wear can already be considered in the case of MoO_3_. For Mo, on the other hand, no wear particles could yet be detected tactilely on the surface. Therefore, the wear fields of Mo were examined by means of SEM to determine possible particles. The wear behavior of both layers correlated with the hardness values determined.

**Figure 12:**
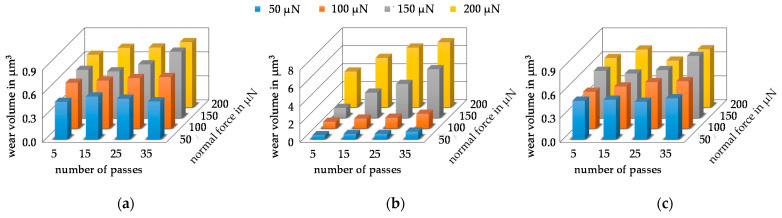
Average values determined of the wear volume of the respective parameter combination as (**a**) volume determination via subtraction of post-scan and pre-scan of Mo-coating. (**b**) Volume determination via subtraction of post-scan and pre-scan of MoO_3_-coating. (**c**) Volume determination via post-scan of Mo-coating only.

To determine the wear volume, the pre-scans and the post-scans were aligned as described in Section 2.3, subtracted from each other and integrated over the wear field to determine the wear volume. In Figure 12a,b, the mean values of the wear volume of the respective parameter combination are plotted as bars. Here, the Mo-layer, in Figure 12a, showed a linear increase in wear volume with rising force as well as an increase in the number of passes. This behavior is consistent with the observation of the wear fields in Figure 11a. The wear volume increased from 0.46 µm^3^ to 0.81 µm^3^. In contrast, the development of the wear volume for the MoO_3_ is shown in Figure 12b. Compared to the Mo-coating, the wear volume was significantly higher, which was to be expected due to the lower hardness. In addition, the wear volume increased exponentially from 150 µN, which indicates a changed wear mechanism. The smallest wear volume of MoO_3_ at 50 µN/5 P was 0.41 µm^3^ and was even slightly smaller than the smallest wear volume of Mo with 0.46 µm^3^. The fact that the volumes are similar, despite the same parameter combination, is a further indication that the surface tensions of the MoO_3_ initially have a wear-reducing effect. After exceeding the surface tensions, the wear volume increased strongly until the maximum was reached with 7.21 µm^3^ at 200 µN/35 P. The larger wear volume was caused by a stronger abrasive wear, which can be seen in Figure 11b in the levelled surfaces and the large material build-up. For the determination of the wear volume in Figure 12c, only the wear field of the post-scan was integrated without taking the pre-scan into account. The results clearly represent the error that occurs when the initial area is not taken into consideration. In particular, in the force series of 200 µN, even the averaged wear volume showed that a discontinuous volume development occurred. That the wear volumes were not completely different is due to the fact that, depending on whether there is a groove or an accumulation of material in the pre-scan, the wear volume determined from the post-scan can sometimes be larger and sometimes smaller, which balances out accordingly on average. This effect can also be seen in the larger standard deviations, which can be seen in Appendix A. There, the wear volumes with standard deviation are listed for both coatings and both evaluation methods.

**Figure 13:**
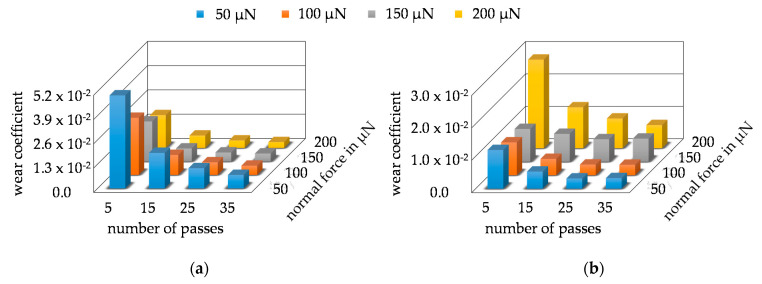
Development of the calculated average values of the coefficient of wear of the respective parameter combination as a 3D bar chart: (**a**) Mo-coating; (**b**) MoO_3_-coating.

The CoW was calculated from the average wear volumes taking into account the layer hardness, the CoF, the respective normal force, and wear paths. Due to the use of the average wear volume, an average CoW was also calculated for the respective parameter combination. The CoW of Mo are plotted in Figure 13a and showed an exponential development contrary to the volume development of Mo. However, the larger CoW for the series with 5 P and for the series with a force of 50 µN can be explained by the linear increase in Mo-wear volume. Since the forces and the number of passes partially double and the volume does not change significantly, the CoW must represent the changes in the forces and the number of passes in order to determine the correct volume. The CoW of MoO_3_ are shown in Figure 13b. Due to the exponential growth of the wear volume of MoO_3_, almost constant CoW were calculated. The slightly increased CoW of the force series 150 µN and 200 µN represent the changed wear behavior of the layer as their volume increased very strongly. Basically, the series with 5 P very well showed the relation between wear volume, CoW, and force. The CoW were almost the same within this series from 50 µN to 150 µN because the wear volume changes in the same proportion as the forces. For example, the wear volume of 50 µN/5 P doubled at a wear volume of 100 µN/5 P and tripled at 150 µN/5 P. At 200 µN/5 P, the force was quadrupled, but the wear volume increased tenfold, which caused the CoW to increase significantly. In order to determine the dependence between wear coefficient, wear volume, force, and number of passes, Appendix B contains further diagrams for both coatings, which show the direct dependency between wear volume, wear coefficient, and the parameter combinations. A comparison of the absolute values of the CoW between the two materials is not practical as the specific layer hardness and the determined CoF of the layer were included in the calculation.

**Figure 14:**
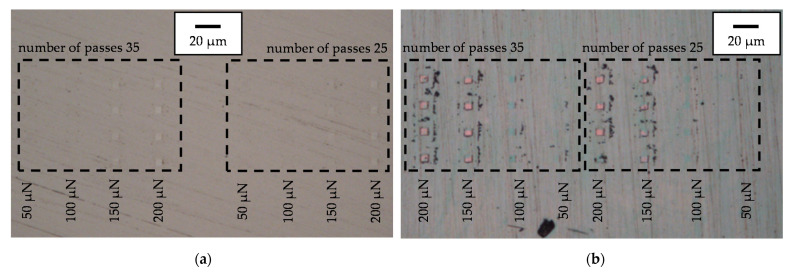
Light microscope images of the measuring locations for wear patches of the series with 35 P and 25 P: (**a**) For the Mo-coating; (**b**) For the MoO_3_-coating.

The wear fields of Mo are clearly visible as bright squares at 150 µN and 200 µN in the light microscopic images in Figure 14a. For a force of 50 µN and 100 µN, the wear fields were not visible, but the reference indents can be recognized there. In addition to the reference indents and the wear fields, it was not possible to see whether particles were removed from the surface of Mo. The wear areas of MoO_3_ are shown in Figure 14b. There, it was first noticeable that the wear fields at 150 µN and 200 µN appeared in pink and those at 100 µN appeared green/blue. The different colors were caused by the different reflection and refraction of the light beam of the optics. Pure Mo is translucent, which is why the wear areas only become visible when the surface is scratched enough and then only as bright squares. In the case of MoO_3_, the black dots that were collected at an even distance around the wear fields were also noticeable. The size and quantity of the black dots increased with increasing force and number of passes. Due to the even spacing of the dots around the wear patches, these can only be particles that have detached from the surface during the wear test and have subsequently been displaced by the post-scan. The larger amount of particles goes hand in hand with the increase in wear volume. Smaller particles can also be seen around the wear fields with 50 µN and 100 µN, so abrasive wear was already present there. In addition, the reference indents of MoO_3_ were not recognizable, because at 4000 µN, they were too small to leave an impression for optical perception.

**Figure 15:**
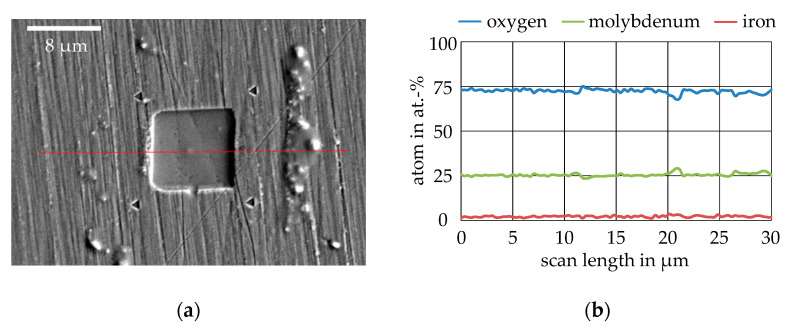
SEM examination of the wear field: (**a**) BSE image of a wear field with 200 µN/35 P on MoO_3_ and course of the line scan; (**b**) Result of the line scan from (**a**).

In order to determine whether particles were detached from the surface during the wear test on the Mo coating, all specimens were examined using SEM. Figure 15a shows a backscattered electron (BSE) image of a wear field of MoO_3_ generated with 200 µN/35 P. The wear field was clearly defined and showed only two slightly indicated furrows on the original surface structure. Particles could be seen at the left edge of the wear field having some adhesion to the surface, as these particles were not pushed to the sides by the post-scan such as the large accumulation of material to the right of the wear field. The particle accumulation on the right in Figure 15a is shown as the black dots that were already visible in the optical image in Figure 14b. The coating showed no delamination at the wear edges or cracking to the reference indents at the corner points of the wear field. White areas surrounding the reference indents were present, which indicate the first slight pile-ups. The red arrow indicates the length and direction of the line scan, which is shown in the diagram in Figure 15b. In the evaluation of the line scan, the elements oxygen, molybdenum, and iron were selected. It can be seen that the three elements showed almost constant values over the entire line scan. Oxygen was at 75 at.%, molybdenum at 25 at.%, and iron was almost undetectable. From the distribution of oxygen and molybdenum, it was confirmed that from the MoO_3_ target, MoO_3_ could also be deposited on the surface. Between 12 µm and 21 µm scan length, small deflections or dips could be seen for oxygen and molybdenum, which marked the transitions from the wear field to the unworn surface. In between, both curves were constant again and at the same level. This indicates that no tribo-oxidation took place within the wear field of molybdenum trioxide and that the wear particles at the end of the line scan did not show any major differences in composition.

**Figure 16:**
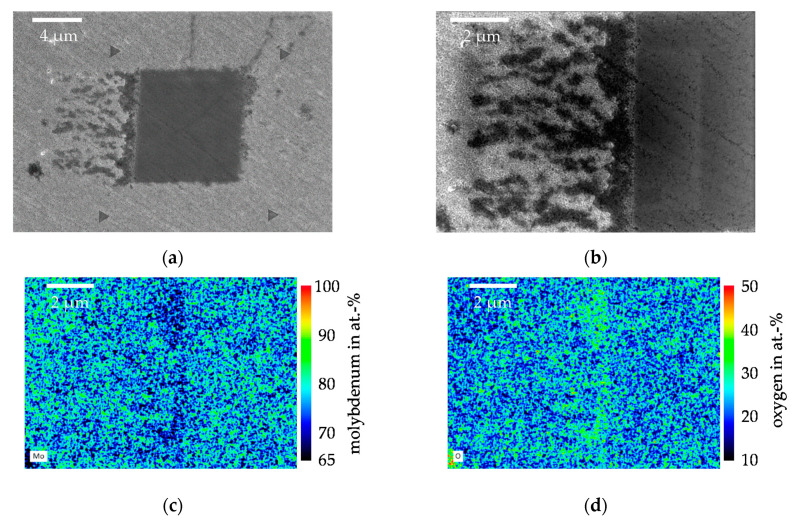
SEM examination of the wear field. (**a**) Secondary electron (SE) image of a wear field with 200 µN/35 P on Mo; (**b**) Enlarged SE image of the left region of the wear field of (**a**) over which the element mapping was performed; (**c**) Result of the mapping from (**b**) for Mo; (**d**) Result of the mapping from (**b**) for oxygen.

In Figure 16a, a secondary electron (SE) image of a Mo wear field generated with 200 µN/35 P is shown. The SE image is necessary because the wear depths in the Mo are very shallow and therefore the wear field can only be detected by an image close to the surface. Once again, the wear field was clearly recognizable due to the darker coloring. The reference indents were also clearly recognizable and showed no pile-ups or cracks toward the wear field. At the edges of the wear field, irregular dark spots were visible, which were very pronounced at the left edge. These areas show particles that have been detached from the surface and subsequently carried away from the wear surface by the post-scan. The particles were very fine and settled between the crystalline surface structures of the Mo. An enlargement of the area of the particles and the left edge of the wear field is shown in Figure 16b. Within the wear field, the original structure could still be seen, but the fine crystallites of the molybdenum had been worn away and partially levelled. In the area of the particles, the crystallites were still intact and showed no wear. In order to assess whether these were actually abrasion particles or whether the chemical composition had changed due to tribo-oxidation, element mapping was carried out over the area in Figure 16b. Figure 16c shows the result of the mapping for Mo and Figure 16d for the mapping of oxygen. There was no increased oxygen or reduced Mo content in the wear surface. However, the particles showed an increased oxygen content and a reduced Mo content. These increases in oxygen content, nevertheless, were still far from those of the MoO_3_ composition. The element mapping showed that no constant oxide layer formed within the wear field, but the wear particles will enrich with oxygen.

### 3.3. Wear Modeling and Validation

**Figure 17:**
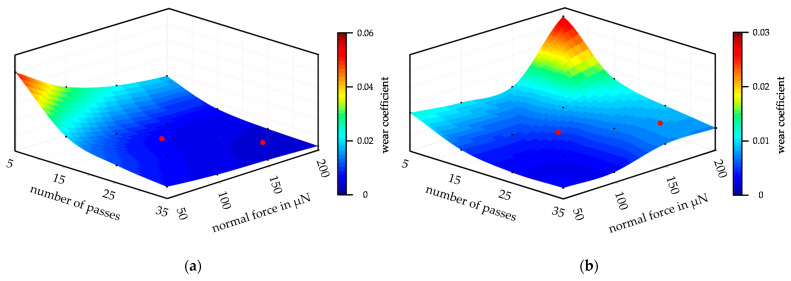
3D plot of the interpolation of the coefficients of wear, black dots represent the reference points and red dots show the values calculated for validation: (**a**) Mo-coating; (**b**) MoO_3_-coating.

In order to be able to predict the wear volume, the wear coefficients determined from the wear test were required. The wear model was valid for the investigated parameter range from 5 P to 35 P and from 50 µN to 200 µN. For the modeling of the CoW, 16 reference points per coating were available. The grid points were interpolated in MATLAB^®^ using a cubic approach so that the CoW between the grid points could also be calculated. The result of the interpolation and the grid points are shown in Figure 17a for the CoW of the Mo and in Figure 17b for the MoO_3_. The black dots represent the grid points from the wear tests, while the color plot shows the interpolation.

**Figure 18:**
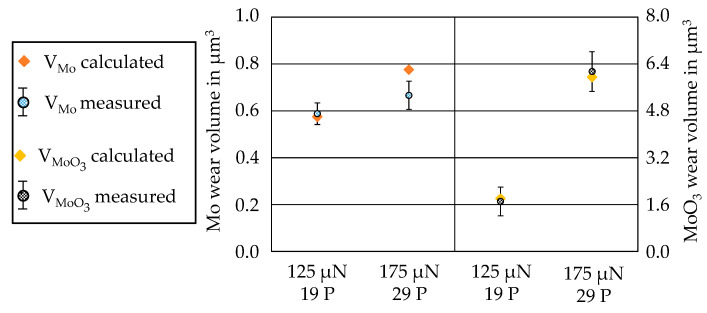
Comparison of the predicted and the experimentally determined wear volumes for Mo and MoO_3_.

By entering the number of passes and the force, the interpolated CoW can be determined and the wear volume can be calculated by the MATLAB^®^ script with the help of the deposited layer hardness and the CoF. In order to validate the wear model, wear tests were carried out with two different parameter combinations, which were both not used for parameterizing the wear model. For each layer, eight tests were carried out with the parameter combination 175 µN/29 P as well as with 125 µN/19 P. CoW calculated from the interpolation of the two parameter combinations are marked as red dots in Figure 17. The calculated CoW can now be used to predict the wear volume for the respective layer and parameter combination. In Figure 18, the predicted wear volumes and the experimentally determined wear volumes are compared. The wear volumes for the Mo-coating match the parameter combination 125 µN/19 P. However, the predicted wear volume for 175 µN/29 P was outside the standard deviation of the experimentally determined wear volumes. The reason for this could be that the wear volume of 150 µN/35 P was slightly larger than that of 200 µN/35 P. This resulted in a larger CoW, which of course influences the interpolation. In addition, this is still an interpolation, which will gain in accuracy with a larger number of grid points. For the MoO_3_-coating, the calculated wear volumes and the mean values of the measured wear volumes were very close to each other. The good agreement of the volumes for both parameter combinations indicates that the wear model can also represent the change in the wear behavior of the MoO_3_ at a normal force of 150 µN very well. Therefore, the developed wear model can be considered successfully validated. With the validated wear model, the wear volume could now be determined for different loads and wear paths within the parameter space considered for the respective coating. In addition, a coupling of the wear models of the Mo- and MoO_3_-coating is conceivable, so that the wear can be predicted with known coating thicknesses in the coating system.

## 4. Conclusions

In this study, a holistic approach to modeling nano wear according to Sarkar’s extended wear model was presented. The determination of the wear volume from nano wear tests was first standardized and automated. By using reference indents and the developed evaluation routine in Gwyddion^®^ and MATLAB^®^, the approach for wear-volume determination according to Wang et al. could be extended and improved [33]. The newly developed method for wear modeling was carried out on the Mo- and MoO_3_-coatings and their wear behavior was analyzed with regard to the requirements for the individual coatings.

By means of the wear investigations, a linear wear behavior was determined for the Mo-coating, which increased continuously with an increase in the force as well as the number of passes. The wear behavior of the MoO_3_-coating showed an exponential increase beginning with a force of 150 µN. Optical analyses and SEM examinations showed abrasive wear in the form of particles for both specimens. The analysis of the wear fields and wear particles showed that the chemical composition of the MoO_3_ did not change due to the tribo-oxidation. From the element mapping, it could be derived for the Mo-coating that within the wear field, no new oxide layer was formed, but the wear particles became more enriched with oxygen. The characterization of the individual layers means the following for the requirements of the entire coating system:

- Rapid abrasion and providing MoO_3_ in the run-in phase due to the low hardness and exponential wear;
- Wear-resistant Mo reservoir due to the high hardness; and
- Regenerative lubrication, presumably only transfer lubrication, of the wear particles.

The wear models developed were successfully validated for the considered parameter range by means of experimental tests. Within the parameter space considered, the wear volume can be calculated with the models. Mo and MoO_3_ feature opposite wear behavior, which should be advantageous for a later application as a dry lubricant on bearing surfaces. Under tribological load such as cyclic over-rolling, both materials can work together efficiently.

## Figures and Tables

**Table 1:** Measurement settings for the in-situ SPM for the pre-scan, the post-scan and the wear field.

Scans	Scan Rate in Hz	Scan Size in µm	Scan Direction	Number of Lines	Scanning Force in µN	Number of Passes (P)
Pre-scan	0.5	20 × 20	horizontal	512	2	1
Wear field	1.0	8 × 8	horizontal	512	50, 100, 125, 150, 175, 200	5, 15, 19, 25, 29, 35
Post-scan	0.5	20 × 20	horizontal	512	2	1

**Table 2:** Test matrix for the wear test for modeling and validation of the wear model with repetition (R) and measuring point (MP).

Number of Pass (P)	Normal Force in µN					
	50	100	150	200	125	175
5	4 R × 2 MP	4 R × 2 MP	4 R × 2 MP	4 R × 2 MP		
15	4 R × 2 MP	4 R × 2 MP	4 R × 2 MP	4 R × 2 MP		
25	4 R × 2 MP	4 R × 2 MP	4 R × 2 MP	4 R × 2 MP		
35	4 R × 2 MP	4 R × 2 MP	4 R × 2 MP	4 R × 2 MP		
19					4 R × 2 MP	
29						4 R × 2 MP

**Table 3:** Average values of the nano hardness, the Young’s modulus, and the CoF for the Mo and MoO_3_ coating and the steel substrate.

Specimen	Nano Hardness in GPa	Standard Deviation	Young’s Modulus in GPa	Standard Deviation	CoF	Standard Deviation
Mo	9.82	0.58	218.10	7.85	0.13	0.03
MoO_3_	2.62	0.17	67.26	2.40	0.20	0.04
Steel substrate ^1^	9.30	0.30	231.00	8.00	-	-

^1^ Results from [34].

**Figure A1:**
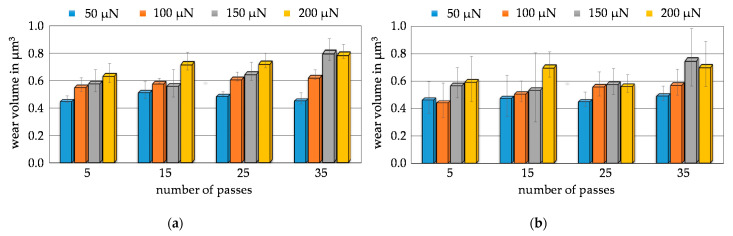
Determined wear volumes with standard deviation of the respective parameter combination. (**a**) Volume determination via subtraction of post-scan and pre-scan of the Mo-coating. (**b**) Volume determination only via post-scan of the Mo-coating.

**Figure A2:**
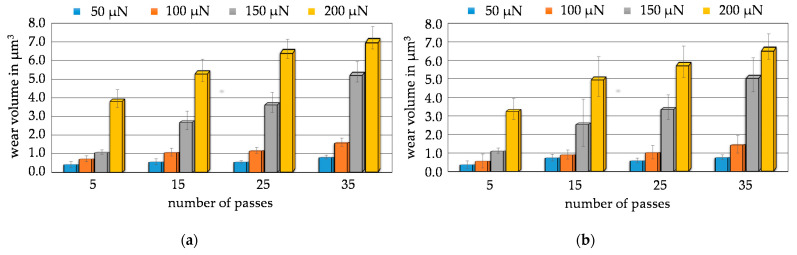
Determined wear volumes with standard deviation of the respective parameter combination. (**a**) Volume determination via subtraction of post-scan and pre-scan of the MoO_3_-coating. (**b**) Volume determination only via post-scan of the MoO_3_-coating.

**Figure A3:**
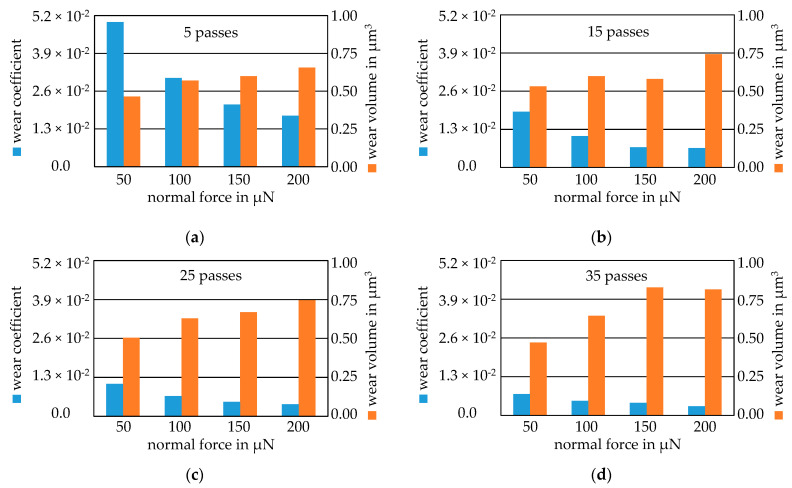
Development of the average wear volumes and average coefficients of wear over the normal forces of the Mo-coating. (**a**) 5 passes; (**b**) 15 passes; (**c**) 25 passes; (**d**) 35 passes.

**Figure A4:**
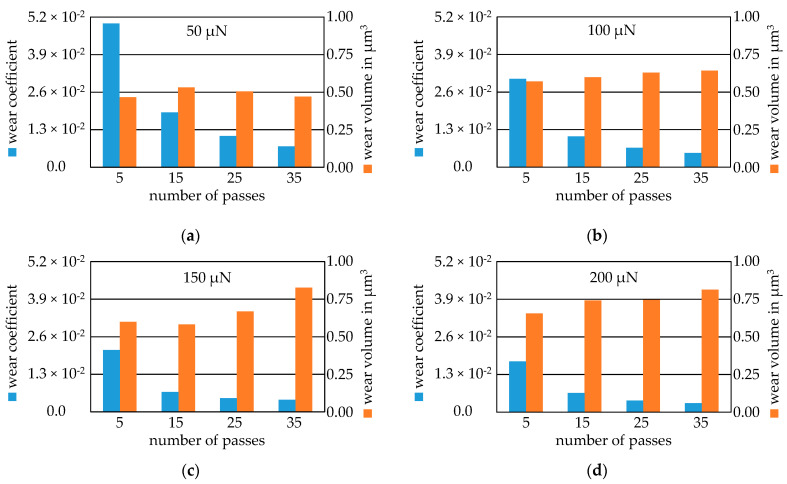
Development of the average wear volumes and average coefficients of wear over the number of passes of the Mo-coating: (**a**) 50 µN; (**b**) 100 µN; (**c**) 150 µN; (**d**) 200 µN.

**Figure A5:**
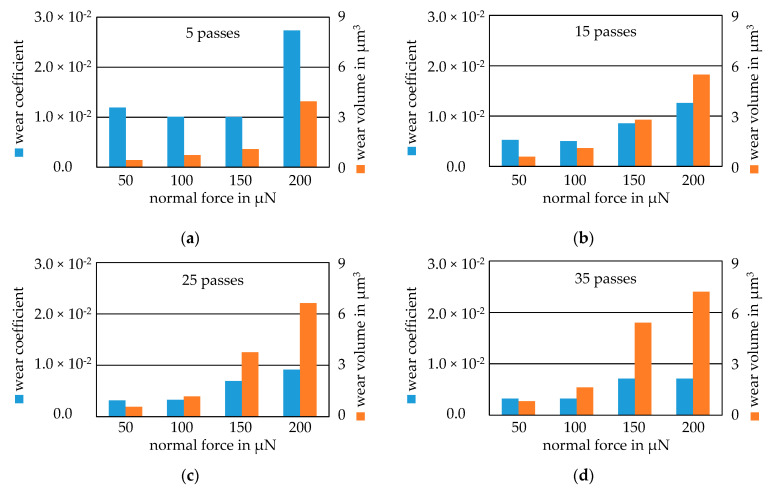
Development of the average wear volumes and average coefficients of wear over the normal forces of the MoO_3_-coating: (**a**) 5 passes; (**b**) 15 passes; (**c**) 25 passes; (**d**) 35 passes.

**Figure A6:**
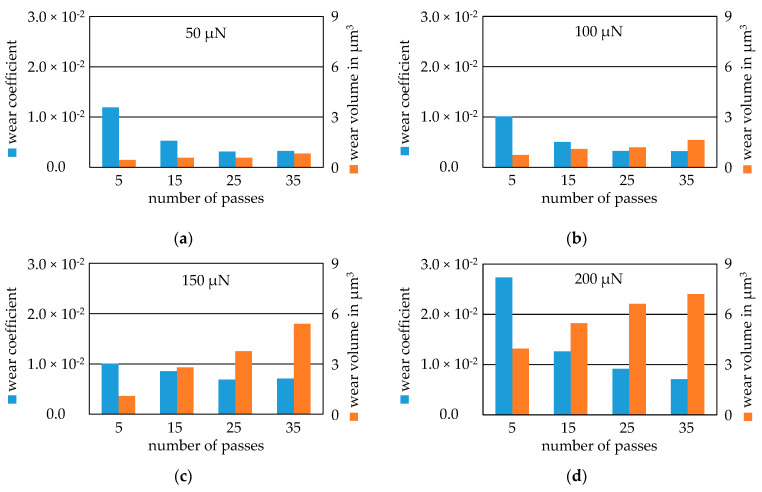
Development of the average wear volumes and average coefficients of wear over the number of passes of the MoO_3_-coating: (**a**) 50 µN; (**b**) 100 µN; (**c**) 150 µN; (**d**) 200 µN.

## Data Availability

The data presented in this study are available on request from the corresponding author.
